# Effect of Weight Class on Regional Brain Volume, Cognition, and Other Neuropsychiatric Outcomes among Professional Fighters

**DOI:** 10.1089/neur.2020.0057

**Published:** 2021-03-18

**Authors:** Michael J.C. Bray, Jerry Tsai, Barry R. Bryant, Bharat R. Narapareddy, Lisa N. Richey, Akshay Krieg, William Tobolowsky, Sahar Jahed, Guogen Shan, Charles B. Bernick, Matthew E. Peters

**Affiliations:** ^1^ Department of Psychiatry and Behavioral Sciences, Johns Hopkins University School of Medicine, Baltimore, Maryland, USA.; ^2^ Department of Psychiatry, Institute of Living, Hartford Hospital, Hartford, Connecticut, USA.; ^3^ Department of Psychiatry and Behavioral Medicine, Medical College of Wisconsin, Milwaukee, Wisconsin, USA.; ^4^ Department of Epidemiology and Biostatistics, School of Public Health, University of Nevada Las Vegas, Las Vegas, Nevada, USA.; ^5^ Department of Neurology, University of Washington, Seattle, Washington, USA.; ^6^ Cleveland Clinic Lou Ruvo Center for Brain Health, Las Vegas, Nevada, USA.

**Keywords:** chronic traumatic encephalopathy, fighting, martial arts, neurodegeneration, neuropsychiatry, traumatic brain injury, weight class

## Abstract

Traumatic brain injury (TBI) is a common source of functional impairment among athletes, military personnel, and the general population. Professional fighters in both boxing and mixed martial arts (MMA) are at particular risk for repetitive TBI and may provide valuable insight into both the pathophysiology of TBI and its consequences. Currently, effects of fighter weight class on brain volumetrics (regional and total) and functional outcomes are unknown. Fifty-three boxers and 103 MMA fighters participating in the Professional Fighters Brain Health Study (PRBHS) underwent volumetric magnetic resonance imaging (MRI) and neuropsychological testing. Fighters were divided into lightweight (≤139.9 lb), middleweight (140.0–178.5 lb), and heavyweight (>178.5 lb). Compared with lightweight fighters, heavyweights displayed greater yearly reductions in regional brain volume (boxers: bilateral thalami; MMA: left thalamus, right putamen) and functional performance (boxers: processing speed, simple and choice reaction; MMA: Trails A and B tests). Lightweights suffered greater reductions in regional brain volume on a per-fight basis (boxers: left thalamus; MMA: right putamen). Heavyweight fighters bore greater yearly burden of regional brain volume and functional decrements, possibly related to differing fight dynamics and force of strikes in this division. Lightweights demonstrated greater volumetric decrements on a per-fight basis. Although more research is needed, greater per-fight decrements in lightweights may be related to practices of weight-cutting, which may increase vulnerability to neurodegeneration post-TBI. Observed decrements associated with weight class may result in progressive impairments in fighter performance, suggesting interventions mitigating the burden of TBI in professional fighters may both improve brain health and increase professional longevity.

## Introduction

Traumatic brain injury (TBI) is increasingly recognized as not only an acute injury event but, in some, also as a progressive neurodegenerative process.^1–3^ As such, it is unsurprising that TBI has been observed to influence neuropsychiatric functioning in the context of neurodegeneration, affecting both symptomatic presentation and clinician evaluation of patients.^4,5^ In the context of contact sports, multiple authors have noted neurodegenerative changes due to TBI with accompanying neuropsychiatric deficits, such as those observed in chronic traumatic encephalopathy (CTE).^6–8^ Although some of the earliest evidence of this was observed in combat sports such as boxing,^9,10^ similar neurological effects of repetitive head impacts (RHIs) have been observed in military personnel and athletes playing football, soccer, and hockey.^11,12^ Boxers and mixed martial arts (MMA) fighters, who regularly receive strikes to the head during practice and competitions, represent important populations at risk for developing neuropsychiatric complications from RHI.^6,8,13^

Boxing and MMA matches have traditionally utilized weight classes to separate competitors into different divisions, such that neither competitor has an unfair physical advantage during a fight. Although fighters need not fight at the exact same weight class in each professional bout, it is uncommon for their fighting weight to deviate substantially throughout their career. Previous findings on association between weight class and injury have been mixed. Higher rates of injury have been documented in heavyweight Muay Thai^14^ and MMA^15^ fighters, whereas other studies showed no difference in risk of injury by weight in boxing^16^ and MMA.^17^ Prior analyses have found differences in fight dynamics by weight division, such as fewer but stronger strikes in heavyweight fighters^18,19^ and more time spent in high-intensity combat situations in lightweight fighters.^20^ However, few studies have examined the effect of weight class on longitudinal changes subsequent to RHI, such as regional brain atrophy, cognitive decline, and behavioral changes.

The purpose of this study was to investigate the relationship between weight class and longitudinal neuroimaging, cognitive testing, and neuropsychiatric outcomes among boxers and MMA fighters participating in the Professional Fighters Brain Health Study (PFBHS).^13^ Under the research-guided assumption that heavier-weight-class fighters sustain a more substantial burden of RHI, we hypothesized that heavier-weight-class fighters would have greater reductions in regional brain volume and worse performance on cognitive and neuropsychiatric measures compared with lighter-weight-class fighters. Specifically, volumetric decline was predicted to be associated with heavier weight class in regions previously identified as vulnerable to volumetric change in TBI and RHI, including the thalamus, putamen, caudate, amygdala, hippocampus, lateral ventricles, and anterior/middle/posterior corpus callosum.^21–23^ Likewise, functional declines were predicted to be associated with heavier weight class in outcomes previously found to be affected by TBI and RHI, including verbal memory, psychomotor speed, processing speed, reaction time, daytime sleepiness, depressive symptoms, and impulse control.^24–28^

## Methods

### Study population

Participants were drawn from the PFBHS, a longitudinal prospective cohort study of professionally licensed boxers and MMA fighters followed at the Cleveland Clinic Luo Ruvo Center for Brain Health, Las Vegas, Nevada starting from 2011, as described previously.^13^ The study was approved by the Cleveland Clinic Institutional Review Board, and participants provided written informed consent. Licensed professional boxers and MMA fighters were recruited through advertisement on the Nevada Athletic Commission website and direct correspondence with managers, promoters, trainers, and fighters.

Inclusion criteria included current license in professional fighting at time of data collection, age ≥18 years, fourth-grade reading proficiency or greater, fluency in English or Spanish, and ability to undergo 3-T brain magnetic resonance imaging (MRI). Exclusion criteria included history of sanctioned professional or amateur fight within 45 days of baseline visit; the rationale for this was to allow time for participants to re-equilibrate from any acute injuries from matches, such as concussion and contusion, to better characterize chronic trends in neuroimaging, cognition, and neuropsychiatric symptoms.^13^ After an initial baseline visit, participants were re-evaluated annually for at least 4 years, with each visit occurring at least 45 days after their most recent sanctioned fight. Demographic information and professional fighting history were obtained from published online records (using online records from the Boxing Records Archive [BoxRec] official website for boxing and from SherDog and MixedMartialArts LLC for MMA). Structural neuroimaging, cognitive, and other neuropsychiatric outcomes were obtained at each visit as follows.

### Neuroimaging

Brain imaging was performed on all fighters at each study visit with a MAGNETOM Verio 3-T scanner (Siemens, Erlangen, Germany) with a 32-channel head coil. Structural, three-dimensional (3D), T1-weighted, magnetization-prepared rapid acquisition gradient echo images were acquired using the following sequence: repetition time 2300 msec/echo time 2.98 msec; resolution 1 mm × 1 mm × 1.2 mm. T1-weighted images were analyzed with the open-source brain image processing program FreeSurfer (version 6.0).^29^ Total brain volume and volumes of specific brain regions including the thalamus, putamen, caudate, amygdala, hippocampus, lateral ventricles, and anterior/middle/posterior corpus callosum were segmented using FreeSurfer's automated full-brain segmentation process following image acquisition. Only images with a signal-to-noise ratio ≥16 and identified as having high-quality cortical reconstruction by FreeSurfer's quality analysis tools were included for analysis. This signal-to-noise ratio of 16 was selected based on pilot observations from randomly selected participants demonstrating that no manual corrections were required with a signal-to-noise ratio ≥16, as previously reported in this population.^30^

### Cognitive function

Cognitive function was assessed using the CNS Vital Signs neurocognitive assessment program.^31^ Results from verbal memory, finger tapping, symbol digit coding, and Stroop tests were used to calculate scores in four neurocognitive domains: verbal memory, psychomotor speed, processing speed, and reaction time; the scores are standardized with a mean of 100 and standard deviation of 15, with higher scores indicative of better performance. Additionally, iComet C3 Logix, a tablet-based application,^32^ was used to measure processing speed, simple reaction time, choice reaction time, visual attention/task switching/neuromotor function (using Trails A and B tests), and balance (Balance Error Scoring System, BESS). Trails A and B were measured in seconds needed to complete the modules, whereas simple reaction time and choice reaction time were measured in milliseconds; greater time is indicative of worse performance. Processing speed was measured as number of correct responses. BESS score represents the number of errors committed during balancing tasks, with higher values indicative of worse performance.

### Neuropsychiatric outcomes

The Patient Health Questionnaire-9 (PHQ-9),^33^ a nine-question self-report tool, was used to screen for symptoms of depression, whereas the Barratt Impulsiveness Scale (BIS-11),^34^ a 30-item questionnaire, was used to characterize first order (attention, cognitive instability, motor, perseverance, self-control, cognitive complexity) and second order (attentional, motor, non-planning) components of impulsiveness. The Epworth Sleepiness Scale (ESS),^35^ an eight-item questionnaire, was used to check for problematic somnolence in everyday situations that may occur in patients with TBI.^36^

### Statistical analysis

Weight class was divided into three categories: light (≤139.9 lb), middle (140.0–178.5 lb), and heavy (>178.5 lb), whereas fight exposure was operationalized as total number of professional fights. Comparison of participant characteristics between boxers and MMA fighters were performed with chi-squared tests for categorical variables (weight class, sex, race) and one-way analysis of variance for continuous variables (number of fights, years of professional fighting, age, years of education). Multiple linear regression models were used to evaluate the role of weight class and fight exposure on changes in regional brain volume, cognition, and neuropsychiatric outcomes with boxers and MMA fighters being analyzed separately. The key predictor variables of interest included in multiple linear regression modeling included weight class, number of fights, and their interaction. Age, sex, race, years of education, and years of professional fighting were selected as covariates *a priori* to control for potential differences in demographics and fighting experience between fighters of different weight classes. All multiple linear regression models were constructed with the lightweight class (≤139.9 lb) as the reference group. In analyses where regional brain volume was the primary outcome variable, multiple regression models also included estimated total intracranial volume as a covariate. All outcome variables of multiple linear regression models, including regional brain volumes and cognitive/neuropsychiatric measures, were expressed in terms of yearly rate of change, calculated by taking the slope across all visits and accounting for time elapsed between visits.

Baseline neuroimaging, cognitive, and neuropsychiatric measurements were included as covariates in their respective models of yearly change. All analyses were performed with the SAS statistical software (version 9.4; SAS Institute Inc., Cary, NC, USA), with the threshold for significance set *a priori* as α = 0.05. Regarding demographic differences or where specific directional hypotheses were present *a priori*, no statistical corrections for multiple comparisons were performed, consistent with current recommendations in the statistical literature.^37^

## Results

### Participant demographics and characteristics

The study cohort included 53 boxers and 103 MMA fighters (Table 1). Representation of participants among weight classes were similar in boxers and MMA fighters, with most participants in the middleweight class, followed by heavyweight and lightweight classes. There were no statistical differences between boxers and MMA fighters regarding number of fights, years of professional fighting, or age. Male participants constituted the vast majority (around 90%) of both boxers and MMA fighters. On average, MMA fighters had a higher level of education than boxers (median, 14 vs. 12 years, *p* < 0.001). There was significant difference in racial breakdown between boxers and MMA fighters (*p* = 0.0094), with greater representation of African-American participants (32.08% vs. 9.71%) and smaller representation of White (30.19% vs. 49.51%) and Pacific Islander (0.00% vs. 3.88%) participants among boxers compared with MMA fighters.

**Table 1.** Participant Demographics and Characteristics

Demographic characteristic	Boxers,* n* = 53 (33.97%)	MMA fighters,* n* = 103 (66.03%)	df	Statistic (F/X^2^)	*P*-value
Weight class, *n* (%)			2	3.5366	0.171
Light	7 (13.21)	8 (7.77)			
Middle	31 (58.49)	51 (49.51)			
Heavy	15 (28.30)	44 (42.72)			
Number of fights, median (IQR)	15.00 (20.00)	13.00 (16.00)	1	0.00	0.981
Years of professional fighting, Mean (SD)	8.63 (5.02)	8.37 (4.32)	1	0.10	0.750
Age, mean (SD)	34.34 (7.15)	32.87 (4.62)	1	2.39	0.124
Education years, median (IQR)	12.00 (2.00)	14.00 (4.00)	1	15.64	<0.001
Sex, *n* (%)			1	0.64	0.424
Male	49 (92.45)	91 (88.35)			
Female	4 (7.55)	12 (11.65)			
Race, *n* (%)			5	15.23	0.009
African American	17 (32.08)	10 (9.71)			
White	16 (30.19)	51 (49.51)			
American Indian/Alaskan Native	1 (1.89)	2 (1.94)			
Asian	2 (3.77)	4 (3.88)			
Pacific Islander	0 (0.00)	4 (3.88)			
Other	17 (32.08)	32 (31.07)			

df, degrees of freedom; IQR, interquartile range; SD, standard deviation.

### Association between weight class and neuroimaging findings

Multiple linear regression revealed significant associations between weight class and change in regional brain volume that differed between boxers and MMA fighters (Table 2).

**Table 2.** Selected Results of Multiple Linear Regression Examining Yearly Change in Neuroimaging, Cognitive, and Neuropsychiatric Findings

Left thalamus proper	Boxers					MMA fighters				
Predictors	Estimates	SE	df	t	*p*	Estimates	SE	df	t	*P*
Weight[Light]	0 (Ref.)									
Weight[Middle]	-172.43	73.92	39	-2.33	**0.025**	-141.60	86.76	87	-1.63	0.106
Weight[Heavy]	-243.10	91.99	39	-2.64	**0.012**	-194.64	92.66	87	-2.10	**0.039**
Professional Fights	-4.23	2.55	39	-1.66	0.105	-12.07	6.98	87	-1.73	0.087
Professional Fights^*^Weight[Light]	0 (Ref.)									
Professional Fights^*^Weight[Middle]	5.09	3.41	39	1.49	0.144	9.91	7.08	87	1.40	0.165
Professional Fights^*^Weight[Heavy]	9.08	3.84	39	2.36	**0.023**	9.13	7.16	87	1.27	0.206

Right thalamus proper	Boxers					MMA fighters				
Predictors	Estimates	SE	df	t	*p*	Estimates	SE	df	t	*P*
Weight[Light]	0 (Ref.)									
Weight[Middle]	-123.85	66.09	39	-1.87	0.069	88.70	73.94	87	1.20	0.234
Weight[Heavy]	-253.71	82.69	39	-3.07	**0.004**	83.29	79.05	87	1.05	0.295
Professional Fights	-3.83	2.31	39	-1.66	0.105	5.68	5.97	87	0.95	0.344
Professional Fights^*^Weight[Light]	0 (Ref.)									
Professional Fights^*^Weight[Middle]	-0.75	3.11	39	-0.24	0.812	-6.33	6.06	87	-1.05	0.299
Professional Fights^*^Weight[Heavy]	6.36	3.52	39	1.81	0.078	-6.11	6.13	87	-1.00	0.322

Right putamen	Boxers					MMA fighters				
Predictors	Estimates	SE	df	t	p	Estimates	SE	df	t	P
Weight[Light]	0 (Ref.)									
Weight[Middle]	121.06	99.95	39	1.21	0.233	-137.93	45.69	87	-3.02	**0.003**
Weight[Heavy]	141.04	125.62	39	1.12	0.268	-141.95	49.00	87	-2.90	**0.005**
Professional Fights	-0.25	3.19	39	-0.08	0.937	-11.98	3.70	87	-3.24	**0.002**
Professional Fights^*^Weight[Light]	0 (Ref.)									
Professional Fights^*^Weight[Middle]	3.18	4.25	39	0.75	0.458	11.92	3.75	87	3.18	**0.002**
Professional Fights^*^Weight[Heavy]	1.19	4.88	39	0.24	0.808	12.12	3.80	87	3.19	**0.002**

Right hippocampus	Boxers					MMA fighters				
Predictors	Estimates	SE	df	t	p	Estimates	SE	df	t	P
Weight[Light]	0 (Ref.)									
Weight[Middle]	-69.30	34.50	39	-2.01	0.052	-75.60	33.41	87	-2.26	**0.026**
Weight[Heavy]	-62.80	42.60	39	-1.47	0.148	-45.10	35.91	87	-1.26	0.213
Professional Fights	-1.28	1.22	39	-1.05	0.301	-1.93	2.69	87	-0.72	0.474
Professional Fights^*^Weight[Light]	0 (Ref.)									
Professional Fights^*^Weight[Middle]	1.17	1.62	39	0.72	0.473	1.76	2.73	87	0.64	0.522
Professional Fights^*^Weight[Heavy]	3.28	1.77	39	1.85	0.071	0.49	2.77	87	0.18	0.860

Choice reaction time (iComet C3)	Boxers					MMA fighters				
Predictors	Estimates	SE	df	t	p	Estimates	SE	df	t	P
Weight[Light]	0 (Ref.)									
Weight[Middle]	23.28	10.72	28	2.17	**0.038**	19.15	13.43	63	1.43	0.159
Weight[Heavy]	44.88	13.65	28	3.29	**0.003**	22.99	13.77	63	1.67	0.100
Professional Fights	0.23	0.35	28	0.67	0.509	0.86	1.05	63	0.82	0.415
Professional Fights^*^Weight[Light]	0 (Ref.)									
Professional Fights^*^Weight[Middle]	-0.13	0.43	28	-0.30	0.768	-1.07	1.09	63	-0.98	0.330
Professional Fights^*^Weight[Heavy]	-0.57	0.56	28	-1.02	0.318	-1.07	1.07	63	-0.99	0.325

Simple reaction time (iComet C3)	Boxers					MMA fighters				
Predictors	Estimates	SE	df	t	p	Estimates	SE	df	t	P
Weight[Light]	0 (Ref.)									
Weight[Middle]	8.54	5.72	28	1.49	0.146	-4.53	11.24	63	-0.40	0.688
Weight[Heavy]	19.50	7.53	28	2.59	**0.015**	-4.36	11.64	63	-0.37	0.710
Professional Fights	0.22	0.20	28	1.10	0.280	0.33	0.89	63	0.37	0.715
Professional Fights^*^Weight[Light]	0 (Ref.)									
Professional Fights^*^Weight[Middle]	0.14	0.24	28	0.59	0.560	-0.26	0.92	63	-0.29	0.776
Professional Fights^*^Weight[Heavy]	-0.27	0.32	28	-0.83	0.412	-0.27	0.91	63	-0.29	0.770

Processing speed (iComet C3)	Boxers					MMA fighters				
Predictors	Estimates	SE	df	t	p	Estimates	SE	df	t	P
Weight[Light]	0 (Ref.)									
Weight[Middle]	-5.18	1.81	28	-2.86	**0.008**	-2.69	1.92	63	-1.40	0.166
Weight[Heavy]	-7.16	2.42	28	-2.96	**0.006**	-2.51	1.98	63	-1.27	0.209
Professional Fights	-0.03	0.06	28	-0.46	0.652	-0.10	0.15	63	-0.67	0.503
Professional Fights^*^Weight[Light]	0 (Ref.)									
Professional Fights^*^Weight[Middle]	0.01	0.07	28	0.14	0.892	0.11	0.16	63	0.73	0.470
Professional Fights^*^Weight[Heavy]	0.06	0.10	28	0.57	0.573	0.09	0.16	63	0.55	0.581

Trails A (iComet C3)	Boxers					MMA fighters				
Predictors	Estimates	SE	df	t	p	Estimates	SE	df	t	P
Weight[Light]	0 (Ref.)									
Weight[Middle]	-3.04	3.20	28	-0.95	0.349	5.36	1.97	62	2.72	**0.009**
Weight[Heavy]	-6.11	4.25	28	-1.44	0.162	5.11	2.03	62	2.51	**0.015**
Professional Fights	0.04	0.10	28	0.38	0.707	0.08	0.16	62	0.49	0.623
Professional Fights^*^Weight[Light]	0 (Ref.)									
Professional Fights^*^Weight[Middle]	0.09	0.13	28	0.68	0.500	-0.08	0.16	62	-0.51	0.611
Professional Fights^*^Weight[Heavy]	0.09	0.18	28	0.53	0.600	-0.07	0.16	62	-0.46	0.649

Trails B (iComet C3)	Boxers					MMA fighters				
Predictors	Estimates	SE	df	t	p	Estimates	SE	df	t	P
Weight[Light]	0 (Ref.)									
Weight[Middle]	12.09	8.34	26	1.45	0.159	15.85	5.26	59	3.01	**0.004**
Weight[Heavy]	18.01	10.75	26	1.67	0.106	17.55	5.45	59	3.22	**0.002**
Professional Fights	0.11	0.28	26	0.40	0.695	0.69	0.41	59	1.67	0.100
Professional Fights^*^Weight[Light]	0 (Ref.)									
Professional Fights^*^Weight[Middle]	0.05	0.34	26	0.14	0.891	-0.61	0.43	59	-1.42	0.161
Professional Fights^*^Weight[Heavy]	-0.33	0.47	26	-0.70	0.488	-0.70	0.42	59	-1.64	0.106

PHQ-9	Boxers					MMA fighters				
Predictors	Estimates	SE	df	t	p	Estimates	SE	df	t	P
Weight[Light]	0 (Ref.)									
Weight[Middle]	-0.16	0.50	42	-0.31	0.755	-1.84	0.80	89	-2.29	**0.024**
Weight[Heavy]	0.11	0.58	42	0.18	0.857	-1.94	0.83	89	-2.32	**0.023**
Professional Fights	0.01	0.02	42	0.44	0.665	-0.16	0.07	89	-2.41	**0.018**
Professional Fights^*^Weight[Light]	0 (Ref.)									
Professional Fights^*^Weight[Middle]	-0.01	0.02	42	-0.29	0.770	0.17	0.07	89	2.48	**0.015**
Professional Fights^*^Weight[Heavy]	-0.01	0.02	42	-0.52	0.608	0.18	0.07	89	2.74	**0.007**

BIS-11	Boxers					MMA fighters				
Predictors	Estimates	SE	df	t	p	Estimates	SE	df	t	P
Weight[Light]	0 (Ref.)									
Weight[Middle]	-4.25	1.40	41	-3.03	**0.004**	1.34	1.86	90	0.72	0.473
Weight[Heavy]	-3.86	1.62	41	-2.39	**0.022**	1.68	1.92	90	0.88	0.383
Professional Fights	-0.05	0.05	41	-0.93	0.356	0.16	0.15	90	1.04	0.302
Professional Fights^*^Weight[Light]	0 (Ref.)									
Professional Fights^*^Weight[Middle]	0.01	0.06	41	0.13	0.900	-0.13	0.15	90	-0.87	0.388
Professional Fights^*^Weight[Heavy]	-0.01	0.07	41	-0.13	0.899	-0.15	0.15	90	-1.00	0.318

Effects of weight class and number of professional fights are presented. Interaction effects between predictor variables are denoted by asterisks (^*^). Significant effects at the *p <* 0.05 level are bolded. All models presented are corrected for age, sex, race, years of education, and years of professional fighting.

df, degrees of freedom; MMA, mixed martial arts; SE, standard error of the mean.

- Boxers: Relative to lightweight boxers, heavyweight boxers showed greater yearly decrease in thalamic volume (left thalamus β = −243.10 mm^3^/year, *p* = 0.012; right thalamus β = −253.70 mm^3^/year, *p* = 0.004). A similar effect was seen in middleweight boxers, although only in the left thalamus (β = −172.40 mm^3^/year, *p* = 0.025). Middleweight boxers also demonstrated a trend toward yearly decline in right hippocampus volume that approached statistical significance (β = −69.30 mm^3^/year, *p* = 0.052). Significant interaction effects were found between weight class and number of fights regarding left thalamic volume in heavyweight boxers (β = 9.08 mm^3^/year/fight, *p* = 0.023). This positive effect estimate reflects greater decrements among lightweight fighters relative to heavyweights on a per fight basis.
- MMA fighters: Relative to lightweight MMA fighters, heavyweight MMA fighters showed greater yearly decrease in right putamen volume (β = −141.95 mm^3^/year, *p* = 0.005). Middleweight MMA fighters also demonstrated significant yearly decrease in right putamen volume (β = −137.93 mm^3^/year, *p* = 0.003) but additionally showed significant yearly decrease in right hippocampus volume (β = −75.60 mm^3^/year, *p* = 0.026), not seen in heavyweight MMA fighters. Like heavyweight boxers, heavyweight MMA fighters also demonstrated significant yearly decrease in left thalamic volume (β = −194.64 mm^3^/year, *p* = 0.0369). Significant interaction effects were found between weight class and number of fights regarding right putamen volume in heavyweight and middleweight MMA fighters (heavyweight, β = 12.12 mm^3^/year/fight, *p* = 0.020; middleweight, β = 11.92 mm^3^/year/fight, *p* = 0.020).

Yearly changes in all other brain regions (caudate, amygdala, lateral ventricles, anterior/middle/posterior corpus callosum) were not associated with weight class.

### Association between weight class and cognitive function

Distinct patterns of change in cognition attributable to weight class were also observed among boxers and MMA fighters (Table 2).

- Boxers: Relative to lightweight boxers, heavyweight boxers had greater yearly increases in choice reaction time (β = 44.88 msec/year, *p* = 0.003) and simple reaction time (β = 19.50 msec/year, *p* = 0.015) measured by iComet C3, as well as greater yearly decrease in processing speed (β = −7.16 correct responses/year, *p* = 0.006) measured by iComet C3; middleweight boxers showed similar trends in simple reaction time (β = 23.28 msec/year, *p* = 0.038) and processing speed (β = −5.18 correct responses/year, *p* = 0.008) only.
- MMA fighters: Relative to lightweight MMA fighters, both heavyweight and middleweight MMA fighters had greater yearly decrease in performance on the Trails A and B tests, which evaluate visual attention, task switching, and neuromotor function (heavyweight, Trails A β = 5.11 sec/year, *p* = 0.015, Trails B β = 17.55 sec/year, *p* = 0.002; middleweight, Trails A β = 5.36 sec/year, *p* = 0.009, Trails B β = 15.85 sec/year, *p* = 0.004).

Cognitive findings associated with weight class in boxers were not seen in MMA fighters, and vice versa. Yearly changes in other cognitive measures (verbal memory, psychomotor speed, balance) were not associated with weight class.

### Association between weight class and neuropsychiatric outcomes

Weight class was also associated with different trajectories in neuropsychiatric symptoms among boxers and MMA fighters (Table 2).

- Boxers: Relative to lightweight boxers, both heavyweight and middleweight boxers showed yearly decrease in total impulsivity score on the BIS-11 (heavyweight β = −3.86 points/year, *p* = 0.022; middleweight β = −4.25points/year, *p* = 0.004).
- MMA fighters: Relative to lightweight MMA fighters, both heavyweight and middleweight MMA fighters showed yearly decrease of total score on the PHQ-9 depression screening tool (heavyweight β = −1.94 points/year, *p* = 0.023; middleweight β = −1.84 points/year, *p* = 0.024). However, a significant interaction effect was found between weight class and number of professional fights, with heavyweight and middleweight MMA fighters demonstrating exacerbated PHQ-9 score increases with increasing number of fights relative to lightweight fighters (heavyweight β = 0.18 points/year/fight, *p* = 0.007; middleweight β = 0.17 points/year, *p* = 0.015).

Neuropsychiatric findings associated with weight class in boxers were not seen in MMA fighters, and vice versa. Yearly changes in sleepiness measured with the ESS were not associated with weight class.

## Discussion

This prospective cohort study examined the relationship between weight class and neuroimaging, cognitive, and neuropsychiatric outcomes among professional fighters. Of note, an earlier, cross-sectional analysis of a smaller sample within the PFBHS found no association between weight of fighters and baseline regional brain volume.^25^ Building on this, the current longitudinal study found temporal trends in structural, cognitive, and neuropsychiatric outcomes that differed by weight class and type of combat sport. These findings may suggest that, although weight class was not previously found to be a determinant of baseline brain health among professional fighters, it alters the course of neurodegenerative changes over time as RHIs are accumulated. Previously reported null findings from the PFBHS regarding baseline neuroimaging and neuropsychiatric outcomes should be interpreted conservatively given the smaller sample size.

Heavyweight boxers had the greatest yearly decline in thalamic volume and processing speed, followed by middleweight boxers, who similarly showed decline in these measures but to a lesser extent. We previously found that increased fight exposure is associated with decreased thalamic volume and processing speed,^38^ and the presence of the same findings in boxers after controlling for years of professional fighting and number of fights may suggest a weight-dependent mechanism of brain injury. However, in boxers, a significant interaction effect between weight class and number of fights was observed, whereby lightweight fighters experienced greater decreases in thalamic volumes on a per-fight basis, suggesting additional complexity. This is demonstrated by the positive effect estimate found for this interaction effect, which indicates these per-fight decrements were observed in lightweight fighters (the reference group for the present analyses) as compared with heavyweights. The finding of worsening reaction time among heavyweight and middleweight boxers is consistent with the literature surrounding these decrements and head injury^26,39^ and, concerningly, may increase the risk of sustaining greater burden of TBI in future professional bouts.^40^

Neuroimaging and cognitive trends disproportionately affecting heavyweights were also seen in MMA fighters. Like heavyweight boxers, heavyweight MMA fighters showed greater yearly decline in left thalamic volume. Heavyweight and middleweight MMA fighters also demonstrated greater yearly decline in right putamen volume, Trails A score, and Trails B score compared with lightweight MMA fighters. Regarding the right putamen, a significant interaction effect was also observed whereby lightweights demonstrated volumetric reductions on a per-fight basis. Reduction in putamen volume has been associated with bimanual motor impairment,^41^ which may explain our study's concurrent findings of reduced putamen volume and worsening performance on the Trails A and B tests in heavyweight and middleweight MMA fighters. These impairments may produce progressive decrements in fighter performance in competition, suggesting that interventions aimed at maintaining the brain health of professional fighters may increase professional longevity. Mitigation of these decrements may improve fighters' ability to defend against future strikes, subsequently reducing further functional decrements through decreased burden of neurological insult.

The association of heavier weight class with greater decline in regional brain volume and cognitive function among boxers and MMA fighters, even after controlling for number of fights and years of professional fighting, necessitates consideration of factors such as fight dynamics and strike force, which may differ by weight class. Although heavyweight fighters have been observed to attempt fewer strikes during bouts compared with lightweights,^18^ individual strikes from heavier fighters bear greater force.^19^ Both male and female MMA fighters in heavier weight classes have been found to have elevated risk for sustaining knock-outs and technical knock-outs from strikes to the head.^42^ The finding of yearly decline in regional brain volume and cognition among heavyweight and middleweight fighters suggests that the force of individual strikes may influence post-TBI functional outcomes independently from the total number of strikes sustained during professional bouts. However, as the interaction effects suggest, this relationship is complex.

In certain regions (left thalamus in boxers, right putamen in MMA fighters), lightweight fighters demonstrated greater yearly reductions in volume on a per-fight basis. The widespread practice of rapid weight loss through dehydration and caloric restriction among professional fighters to meet weight class requirements^43^ may be more common in lighter-weight-class fighters and underlie their increased susceptibility to brain injury with increasing number of official fights. Although fighters may begin re-feeding and re-hydration immediately after weigh-in, previous studies have found dehydration to be highly prevalent among fighters at the time of competition.^44–46^ Dehydration secondary to rapid cutting of weight may worsen TBI-induced neurotrauma,^47,48^ potentially contributing to the observed association of lighter weight class with worse outcomes on a per-fight basis. This effect would likely only persist during the acute period following dehydration, and as such would only alter the impact of TBIs sustained during professional bouts. It has been proposed that dehydration-induced changes in brain morphology^49,50^ may alter mechanical cushioning and strain mitigation provided in part by cerebrospinal fluid within the subarachnoid space and lateral ventricles.^47,51,52^

Although the exact mechanisms are unclear, the present findings are consistent with this hypothesis and highlight the need for future research into the influence of weight-cutting on the long-term brain health of professional fighters. It should also be considered that heavyweights may have more comorbidities (i.e. diabetes, metabolic syndrome, etc.),^53,54^ potentially exacerbating neurodegenerative processes through pro-inflammatory pathways.^55,56^ This might contribute to greater yearly decrements in heavyweights despite the greater volume of strikes absorbed by lightweights on a per-fight basis and the possible exacerbations from effects of weight-cutting. Future work is required to elucidate these complex relationships.

The finding of greater yearly volumetric decline in the right hippocampi of middleweight MMA fighters may reflect propensities for brain injury inherent to middleweight fights, such as its combination of quality and quantity of strikes. Future studies may examine whether a threshold effect exists for force of strikes in professional fighting, such that significantly greater risk of brain injury is seen after crossing a certain threshold of force.

Contrary to our hypothesis, we found association of heavier weight class with yearly improvement in impulsivity (boxers) and depression (MMA) relative to lightweight fighters. A previous investigation of the PFBHS found that decrease in thalamic volume was associated with lower motor impulsiveness,^57^ which may be related to our findings of yearly decline in thalamic volume and decreased impulsiveness among heavyweight and middleweight boxers in this study. Alternatively, assuming greater brain injury in fighters of heavier weight classes, lower self-reported impulsivity among heavyweight and middleweight boxers may result from reduced insight and underestimation, which has been observed not only in moderate to severe traumatic brain injury,^58^ but also in CTE.^59^

A similar finding was observed for heavyweight and middleweight MMA fighters, who demonstrated yearly improvement in depressive symptoms relative to lightweight MMA fighters despite regional brain volume reductions (left thalamus in heavyweight MMA fighters, right hippocampus in middleweight MMA fighters) that have been previously associated with development of depression following TBI.^28^ Adding to this, significant interaction effects suggested that heavyweight and middleweight fighters experienced greater improvements in depressive symptoms on a per-fight basis. Since multiple regression models were constructed with lightweight status as a reference group, the presence of yearly improvement in impulsivity and depression among heavy/middleweight boxers and MMA fighters, respectively, reflects higher risk of developing behavioral changes among lightweight fighters. Considering that lightweight fighters may engage in weight-cutting more often than their heavyweight counterparts, these findings are consistent with previous studies demonstrating worsening of concentration and depressive symptoms among professional fighters following rapid weight loss.^60^

However, it should be noted that participants in this study had not participated in any professional bouts within 45 days of evaluation, and observed changes in impulsivity and depression likely do not represent acute effects of rapid weight loss and dehydration. Future work may investigate whether intermittent weight-cutting prior to competitions throughout the year is associated with long-term development and persistence of these behavioral symptoms after TBI.

Interestingly, the effect of weight class was not consistent between boxers and MMA fighters, with different decrements in neuroimaging and functional outcomes. These differences may be due to several distinctions between the two combat disciplines, such as use of heavier gloves in boxing, the possible role of ischemic damage from chokes in MMA, or different training and sparring regimens. Boxing and MMA also differ significantly in the range of possible strikes; boxing only allows punches, whereas MMA may involve additional maneuvers such as kicks, elbow strikes, takedowns, and grappling, which introduce an additional layer of complexity in the assessment of traumatic and ischemic brain injury. Additionally, aerobic exercise is associated with improvements in neuropsychiatric functioning and therefore differences in aerobic activity between groups may confound these relationships.^61^ Further research is required to explain these differences and may have the potential to inform understandings of TBI pathophysiology in combat sports, perhaps with implications extending to TBI in the general population as well.

A limitation of this study was the combination of traditional professional fighting weight classes (i.e., flyweight, bantamweight, featherweight, lightweight, welterweight, middleweight, light heavyweight, heavyweight) into three weight classes (light, ≤139.9 lb, middle 140.0–178.5 lb, and heavy, >178.5 lb) to ensure a large enough sample size in each group. The current study also lacked a non-fighting control group that underwent the same tests. Although we controlled for years of professional fighting and number of fights, future investigations may also account for time spent in training, during which fighters may experience repetitive head strikes and chokes contributing to CTE. Although no participants had sanctioned fights within 45 days of study visits, our study did not account for time since their most recent fight, which may be valuable to adjust for in future investigations.

We did not find significant associations between weight class and performance in any of the four CNS Vital Signs neurocognitive domains (Supplementary Table S1). Considering that both CNS Vital Signs and iComet C3 measured processing speed and reaction time but only results from the latter were significantly associated with weight class, future studies may incorporate additional validated instruments for neurocognitive testing. Further, the lack of *a-priori* calculations of sample size in this study may represent a limitation of this investigation. As such, null findings of the present study may indicate lack of adequate statistical power as opposed to true null findings. Lastly, although use of automatic image segmentation has the advantages of reducing variability due to human error or other operator factors and improving replicability of the present study, it may be limited in its ability to control for intra-participant artifacts. Use of a signal-to-noise ratio threshold, established using a pilot sample, likely reduced segmentation error due to noise but it cannot be discounted that participants outside of that pilot sample may represent outliers. Results should therefore be interpreted conservatively.

## Conclusion

Weight class appears to be an important determinant of brain health outcomes in professional fighters, although the relationship is complex and warrants further investigation. The present results may indicate that heavier weight class is associated with increased burden of TBI and its sequelae, although the practice of weight-cutting may increase burden of TBI among lightweights on a per-fight basis, during professional bouts. Understanding differential risks of brain injury among individual weight classes may help organizing bodies, trainers, and professional fighters optimize weight-class-specific regulations, training regimens, and safety equipment.

## Supplementary Material

Supplemental data

## Abbreviations Used

3D: three dimensional
BESS: Balance Error Scoring System
BIS-11: Barratt Impulsiveness Scale
CTE: chronic traumatic encephalopathy
df: degrees of freedom
ESS: Epworth Sleepiness Scale
IQR: interquartile range
MMA: mixed martial arts
MRI: magnetic resonance imaging
PHQ-9: Patient Health Questionnaire-9
PRBHS: Professional Fighters Brain Health Study
RHI: repetitive head impact
SD: standard deviation
SE: standard error of the mean
TBI: traumatic brain injury

## References

[1] Bray, M.J.C. (2018). Examining Causes and Consequences of Mental Health Disorders in Chronic Traumatic Brain Injury. Toronto, Ontario, Canada: University of Toronto. DOI: 10.13140/RG.2.2.35052.72327

[2] Green, R.E.A. (2016). Editorial: Brain injury as a neurodegenerative disorder. Front. Hum. Neurosci. 9, 615 26778994 10.3389/fnhum.2015.00615 PMC4700280

[3] Bigler, E.D. (2013). Traumatic brain injury, neuroimaging, and neurodegeneration. Front. in Hum. Neurosci. 7, 395 10.3389/fnhum.2013.00395 PMC3734373 23964217

[4] Bray, M.J.C., Richey, L.N., Bryant, B.R., Krieg, A., Jahed, S., Tobolowsky, W., LoBue, C., and Peters, M.E. (2021). Traumatic brain injury alters neuropsychiatric symptomatology in all-cause dementia. Alzheimers Dement. DOI: 10.1002/alz.12225 [online ahead of print] PMC8043985 33470043

[5] Bray, M.J.C., Pradeep, T., Arun, S., Richey, L.N., Jahed, S., Bryant, B.R., LoBue, C., Lyketsos, C.G., Kim, P., and Peters, M.E. (2019). History of traumatic brain injury interferes with accurate diagnosis of Alzheimer's dementia: a nation-wide case-control study. Int. Rev. Psychiatry, 1–10 10.1080/09540261.2019.1682529 PMC6952566 31707905

[6] Bryant, B.R., Narapareddy, B.R., Bray, M.J.C., Richey, L.N., Krieg, A., Shan, G., Peters, M.E., and Bernick, C.B. (2019). The effect of age of first exposure to competitive fighting on cognitive and other neuropsychiatric symptoms and brain volume. Int. Rev. Psychiatry, 1–7 10.1080/09540261.2019.1665501 31587599

[7] Gardner, A., Iverson, G.L., and McCrory, P. (2014). Chronic traumatic encephalopathy in sport: a systematic review. Br. J. Sports Med. 48, 84–90 23803602 10.1136/bjsports-2013-092646

[8] McCrory, P., Zazryn, T., and Cameron, P. (2007). The evidence for chronic traumatic encephalopathy in boxing. Sports Med. 37, 467–476 17503873 10.2165/00007256-200737060-00001

[9] Martland, H.S. (1928). Punch drunk. JAMA 91, 1103–1107

[10] Millspaugh, J. (1937). Dementia pugilistica. US Naval Med. Bull. 35

[11] McKee, A.C., Cantu, R.C., Nowinski, C.J., Hedley-Whyte, E.T., Gavett, B.E., Budson, A.E., Santini, V.E., Lee, H.-S., Kubilus, C.A., and Stern, R.A. (2009). Chronic traumatic encephalopathy in athletes: progressive tauopathy after repetitive head injury. J. Neuropathol. Exp. Neurol. 68, 709–735 19535999 10.1097/NEN.0b013e3181a9d503 PMC2945234

[12] McKee, A.C., Stein, T.D., Nowinski, C.J., Stern, R.A., Daneshvar, D.H., Alvarez, V.E., Lee, H.S., Hall, G., Wojtowicz, S.M., Baugh, C.M., Riley, D.O., Kubilus, C.A., Cormier, K.A., Jacobs, M.A., Martin, B.R., Abraham, C.R., Ikezu, T., Reichard, R.R., Wolozin, B.L., Budson, A.E., Goldstein, L.E., Kowall, N.W., and Cantu, R.C. (2013). The spectrum of disease in chronic traumatic encephalopathy. Brain 136, 43–64 23208308 10.1093/brain/aws307 PMC3624697

[13] Bernick, C., Banks, S., Phillips, M., Lowe, M., Shin, W., Obuchowski, N., Jones, S., and Modic, M. (2013). Professional fighters brain health study: rationale and methods. Am. J. Epidemiol. 178, 280–286 23735309 10.1093/aje/kws456

[14] Gartland, S., Malik, M.H., and Lovell, M. (2005). A prospective study of injuries sustained during competitive Muay Thai kickboxing. Clin. J. Sport Med. 15, 34–36 15654189 10.1097/00042752-200501000-00007

[15] Lystad, R.P., Gregory, K., and Wilson, J. (2014). The epidemiology of injuries in mixed martial arts: a systematic review and meta-analysis. Orthop. J. Sports Med. 2, DOI: 10.1177/2325967113518492 PMC4555522 26535267

[16] Zazryn, T.R., McCrory, P.R., and Cameron, P.A. (2009). Injury rates and risk factors in competitive professional boxing. Clin. J. Sport Med. 19, 20–25 19124979 10.1097/JSM.0b013e31818f1582

[17] Ngai, K.M., Levy, F., and Hsu, E.B. (2008). Injury trends in sanctioned mixed martial arts competition: a 5-year review from 2002 to 2007. Br. J. Sports Med. 42, 686–689 18308883 10.1136/bjsm.2007.044891

[18] Miarka, B., Brito, C.J., Bello, F.D., and Amtmann, J. (2017). Motor actions and spatiotemporal changes by weight divisions of mixed martial arts: applications for training. Hum. Move. Sci. 55, 73–80 10.1016/j.humov.2017.07.009 28779598

[19] Walilko, T.J., Viano, D.C., and Viano, D.C. (2005). Biomechanics of the head for Olympic boxer punches to the face. Br. J. Sports Med. 39, 710–719 16183766 10.1136/bjsm.2004.014126 PMC1725037

[20] Miarka, B., Coswig, V.S., Vecchio, F.B.D., Brito, C.J., and Amtmann, J. (2015). Comparisons of time-motion analysis of mixed martial arts rounds by weight divisions. Int. J. Performance Analysis Sport 15, 1189–1201

[21] Schnakers, C., Lutkenhoff, E.S., Bio, B.J., Mcarthur, D.L., Vespa, P.M., and Monti, M.M. (2019). Acute EEG spectra characteristics predict thalamic atrophy after severe TBI. J. Neurol. Neurosurg. Psychiatry 90, 617–619 29954872 10.1136/jnnp-2017-317829 PMC6310668

[22] Green, R.E.A., Colella, B., Maller, J.J., Bayley, M., Glazer, J., and Mikulis, D.J. (2014). Scale and pattern of atrophy in the chronic stages of moderate-severe TBI. Front. Hum. Neurosci. 8, 67 24744712 10.3389/fnhum.2014.00067 PMC3978360

[23] Leunissen, I., Coxon, J.P., Caeyenberghs, K., Michiels, K., Sunaert, S., and Swinnen, S.P. (2014). Subcortical volume analysis in traumatic brain injury: the importance of the fronto-striato-thalamic circuit in task switching. Cortex 51, 67–81 24290948 10.1016/j.cortex.2013.10.009

[24] McAllister, T., and McCrea, M. (2017). Long-term cognitive and neuropsychiatric consequences of repetitive concussion and head-impact exposure. J. Athl. Train. 52, 309–317 28387556 10.4085/1062-6050-52.1.14 PMC5384827

[25] Bernick, C., Banks, S.J., Shin, W., Obuchowski, N., Butler, S., Noback, M., Phillips, M., Lowe, M., Jones, S., and Modic, M. (2015). Repeated head trauma is associated with smaller thalamic volumes and slower processing speed: the Professional Fighters' Brain Health Study. Br. J. Sports Med. 49, 1007–1011 25633832 10.1136/bjsports-2014-093877 PMC4518758

[26] van Zomeren, A.H., and Deelman, B.G. (1976). Differential effects of simple and choice reaction after closed head injury. Clin. Neurol. Neurosurg. 79, 81–90 1029638 10.1016/0303-8467(76)90001-9

[27] Viola-Saltzman, M., and Watson, N.F. (2012). Traumatic brain injury and sleep disorders. Neurol. Clin. 30, 1299–1312 23099139 10.1016/j.ncl.2012.08.008 PMC3482689

[28] Schwarzbold, M., Diaz, A., Martins, E.T., Rufino, A., Amante, L.N., Thais, M.E., Quevedo, J., Hohl, A., Linhares, M.N., and Walz, R. (2008). Psychiatric disorders and traumatic brain injury. Neuropsychiatric Dis. Treat. 4, 797–816 10.2147/ndt.s2653 PMC2536546 19043523

[29] Fischl, B. (2012). FreeSurfer. NeuroImage 62, 774–781 22248573 10.1016/j.neuroimage.2012.01.021 PMC3685476

[30] Bernick, C., Shan, G., Zetterberg, H., Banks, S., Mishra, V.R., Bekris, L., Leverenz, J.B., and Blennow, K. (2020). Longitudinal change in regional brain volumes with exposure to repetitive head impacts. Neurology 94, e232–e240 31871218 10.1212/WNL.0000000000008817 PMC7108810

[31] Gualtieri, C.T., and Johnson, L.G. (2006). Reliability and validity of a computerized neurocognitive test battery, CNS Vital Signs. Arch. Clin. Neuropsychol. 21, 623–643 17014981 10.1016/j.acn.2006.05.007

[32] Borges, A., Raab, S., and Lininger, M. (2017). A Comprehensive Instrument for Evaluating Mild Traumatic Brain Injury (mTBI)/concussion in independent adults: a pilot study. Int. J. Sports Phys. Ther. 12, 381–389 28593091 PMC5455187

[33] Kroenke, K., Spitzer, R.L., and Williams, J.B.W. (2001). The PHQ-9: validity of a brief depression severity measure. J. Gen. Intern. Med. 16, 606–613 11556941 10.1046/j.1525-1497.2001.016009606.x PMC1495268

[34] Patton, J.H., Stanford, M.S., and Barratt, E.S. (1995). Factor structure of the Barratt Impulsiveness Scale. J. Clin. Psychol. 51, 768–774 8778124 10.1002/1097-4679(199511)51:6<768::aid-jclp2270510607>3.0.co;2-1

[35] Johns, M.W. (1991). A new method for measuring daytime sleepiness: the Epworth sleepiness scale. Sleep 14, 540–545 1798888 10.1093/sleep/14.6.540

[36] Verma, A., Anand, V., and Verma, N.P. (2007). Sleep disorders in chronic traumatic brain injury. J. Clin. Sleep Med. 3, 357–362 17694723 PMC1978305

[37] Streiner, D.L., and Norman, G.R. (2011). Correction for multiple testing: is there a resolution? Chest 140, 16–18 21729890 10.1378/chest.11-0523

[38] Bernick, C., and Banks, S. (2013). What boxing tells us about repetitive head trauma and the brain. Alzheimers Res. Ther. 5, 23 23731821 10.1186/alzrt177 PMC3706825

[39] Warden, D.L., Bleiberg, J., Cameron, K.L., Ecklund, J., Walter, J., Sparling, M.B., Reeves, D., Reynolds, K.Y., and Arciero, R. (2001). Persistent prolongation of simple reaction time in sports concussion. Neurology 57, 524–526 11502926 10.1212/wnl.57.3.524

[40] Moriarity, J., Collie, A., Olson, D., Buchanan, J., Leary, P., McStephen, M., and McCrory, P. (2004). A prospective controlled study of cognitive function during an amateur boxing tournament. Neurology 62, 1497–1502 15136671 10.1212/wnl.62.9.1497

[41] Gooijers, J., Chalavi, S., Beeckmans, K., Michiels, K., Lafosse, C., Sunaert, S., and Swinnen, S.P. (2016). Subcortical volume loss in the thalamus, putamen, and pallidum, induced by traumatic brain injury, is associated with motor performance deficits. Neurorehabil. Neural Repair 30, 603–614 26498433 10.1177/1545968315613448

[42] Follmer, B., Dellagrana, R.A., and Zehr, E.P. (2019). Head trauma exposure in mixed martial arts varies according to sex and weight class. Sports Health 11, 280–285 30768376 10.1177/1941738119827966 PMC6537320

[43] Hillier, M., Sutton, L., James, L., Mojtahedi, D., Keay, N., and Hind, K. (2019). High prevalence and magnitude of rapid weight loss in mixed martial arts athletes. Int. J. Sport Nutr. Exerc. Metab. 29, 512–517 31142173 10.1123/ijsnem.2018-0393

[44] Jetton, A.M., Lawrence, M.M., Meucci, M., Haines, T.L., Collier, S.R., Morris, D.M., and Utter, A.C. (2013). Dehydration and acute weight gain in mixed martial arts fighters before competition. J. Strength Cond. Res. 27, 1322–1326 23439336 10.1519/JSC.0b013e31828a1e91

[45] Matthews, J.J., and Nicholas, C. (2017). Extreme rapid weight loss and rapid weight gain observed in UK mixed martial arts athletes preparing for competition. Int. J. Sport Nutr. Exerc. Metab. 27, 122–129 27710145 10.1123/ijsnem.2016-0174

[46] Pettersson, S., and Berg, C.M. (2014). Hydration status in elite wrestlers, judokas, boxers, and taekwondo athletes on competition day. Int. J. Sport Nutr. Exerc. Metab. 24, 267–275 24280038 10.1123/ijsnem.2013-0100

[47] Barley, O., Chapman, D., and Abbiss, C. (2019). The current state of weight-cutting in combat sports. Sports 7, 123 10.3390/sports7050123 PMC6572325 31117325

[48] Crighton, B., Close, G.L., and Morton, J.P. (2016). Alarming weight cutting behaviours in mixed martial arts: a cause for concern and a call for action. Br. J. Sports Med. 50, 446–447 26459278 10.1136/bjsports-2015-094732

[49] Dickson, J.M., Weavers, H.M., Mitchell, N., Winter, E.M., Wilkinson, I.D., van Beek, E.J.R., Wild, J.M., and Griffiths, P.D. (2005). The effects of dehydration on brain volume—preliminary results. Int. J. Sports Med. 26, 481–485 16037892 10.1055/s-2004-821318

[50] Kempton, M.J., Ettinger, U., Schmechtlg, A., Winter, E.M., Smith, L., McMorris, T., Wilkinson, I.D., Williams, S.C.R., and Smith, M.S. (2009). Effects of acute dehydration on brain morphology in healthy humans. Hum. Brain Mapp. 30, 291–298 18064587 10.1002/hbm.20500 PMC6871128

[51] Toma, M., and Nguyen, P.D.H. (2018). Fluid–structure interaction analysis of cerebrospinal fluid with a comprehensive head model subject to a rapid acceleration and deceleration. Brain Injury 32, 1576–1584 30059633 10.1080/02699052.2018.1502470

[52] Ivarsson, J., Viano, D.C., Lövsund, P., and Aldman, B. (2000). Strain relief from the cerebral ventricles during head impact: experimental studies on natural protection of the brain. J. Biomechanics 33, 181–189 10.1016/s0021-9290(99)00144-x 10653031

[53] Guo, J., Zhang, X., Wang, L., Guo, Y., and Xie, M. (2013). Prevalence of metabolic syndrome and its components among Chinese professional athletes of strength sports with different body weight categories. PLoS ONE 8, e79758 24255714 10.1371/journal.pone.0079758 PMC3821854

[54] Murata, H., Oshima, S., Torii, S., Taguchi, M., and Higuchi, M. (2016). Characteristics of body composition and cardiometabolic risk of Japanese male heavyweight Judo athletes. J. Physiol. Anthropol. 35, 10 27048601 10.1186/s40101-016-0092-8 PMC4822258

[55] Boulos, M.E., and Bray, M.J.C. (2018). Complement c3 inhibition modulates neurodegeneration in chronic traumatic brain injury. J. Neurosci. 38, 7201–7203 30111576 10.1523/JNEUROSCI.1011-18.2018 PMC6596038

[56] Rojas-Gutierrez, E., Muñoz-Arenas, G., Treviño, S., Espinosa, B., Chavez, R., Rojas, K., Flores, G., Díaz, A., and Guevara, J. (2017). Alzheimer's disease and metabolic syndrome: a link from oxidative stress and inflammation to neurodegeneration. Synapse 71, e21990 28650104 10.1002/syn.21990

[57] Banks, S.J., Mayer, B., Obuchowski, N., Shin, W., Lowe, M., Phillips, M., Modic, M., and Bernick, C. (2014). Impulsiveness in professional fighters. J. Neuropsychiatry Clin. Neurosci. 26, 44–50 24515676 10.1176/appi.neuropsych.12070185

[58] Rochat, L., Beni, C., Billieux, J., Azouvi, P., Annoni, J.M., and van der Linden, M. (2010). Assessment of impulsivity after moderate to severe traumatic brain injury. Neuropsychol. Rehabil. 20, 778–797 20635306 10.1080/09602011.2010.495245

[59] Mez, J., Stern, R.A., and McKee, A.C. (2013). Chronic traumatic encephalopathy: where are we and where are we going? topical collection on dementia. Curr. Neurol. Neurosci. Rep. 13, 1–12 10.1007/s11910-013-0407-7 PMC4550089 24136455

[60] Franchini, E., Brito, C.J., and Artioli, G.G. (2012). Weight loss in combat sports: physiological, psychological and performance effects. J. Int. Soc. Sports Nutr. 9, 52 23237303 10.1186/1550-2783-9-52 PMC3607973

[61] Bray, M.J.C., Collica, S.C., Peters, M.E., and Tamashiro, K.L. (2020). Exercise interventions for mental health, in: *Johns Hopkins Psychiatry Guide*. M.E. Peters, O.J. Bienvenu, and P. Nestadt. (eds). Johns Hopkins University: Baltimore, MD

