# Supplementary material for: Effect of Weight Class on Regional Brain Volume, Cognition, and Other Neuropsychiatric Outcomes among Professional Fighters
Source: Neurotrauma Rep. 2021 Mar 18;2(1):169–79. doi: 10.1089/neur.2020.0057 (PMC8240832; doi:10.1089/neur.2020.0057)
Supplement: Supplemental data [file Supp_TableS1.docx]

**Supplementary Table S1**. Results of Multiple Linear Regression for CNS Vital Signs Outcomes.

| **Psychomotor speed (CNS Vital Signs)** | **Boxers** |  |  |  |  | **MMA Fighters** |  |  |  |  |
| --- | --- | --- | --- | --- | --- | --- | --- | --- | --- | --- |
| **Predictors** | **Estimates** | **SE** | **df** | **t** | **p** | **Estimates** | **SE** | **df** | **t** | **P** |
| Weight[Light] | 0 (Ref.) |  |  |  |  | 0 (Ref.) |  |  |  |  |
| Weight[Middle] | 1.08 | 3.07 | 41 | 0.35 | 0.726 | -5.29 | 3.52 | 90 | -1.50 | 0.137 |
| Weight[Heavy] | -1.53 | 3.47 | 41 | -0.44 | 0.663 | -3.75 | 3.68 | 90 | -1.02 | 0.311 |
| Professional Fights | 0.07 | 0.10 | 41 | 0.66 | 0.514 | 0.41 | 0.29 | 90 | 1.42 | 0.158 |
| Professional Fights*Weight[Light] | 0 (Ref.) |  |  |  |  | 0 (Ref.) |  |  |  |  |
| Professional Fights*Weight[Middle] | 0.01 | 0.14 | 41 | 0.11 | 0.914 | -0.40 | 0.29 | 90 | -1.38 | 0.171 |
| Professional Fights*Weight[Heavy] | 0.10 | 0.15 | 41 | 0.66 | 0.512 | -0.46 | 0.29 | 90 | -1.58 | 0.119 |
|  |  |  |  |  |  |  |  |  |  |  |
| **Processing speed (CNS Vital Signs)** | **Boxers** |  |  |  |  | **MMA Fighters** |  |  |  |  |
| **Predictors** | **Estimates** | **SE** | **df** | **t** | **p** | **Estimates** | **SE** | **df** | **t** | **P** |
| Weight[Light] | 0 (Ref.) |  |  |  |  |  |  |  |  |  |
| Weight[Middle] | 0.21 | 1.78 | 41 | 0.12 | 0.908 | -2.36 | 2.44 | 90 | -0.97 | 0.336 |
| Weight[Heavy] | -1.37 | 2.07 | 41 | -0.66 | 0.510 | -1.81 | 2.56 | 90 | -0.71 | 0.481 |
| Professional Fights | 0.01 | 0.06 | 41 | 0.08 | 0.934 | 0.09 | 0.20 | 90 | 0.44 | 0.662 |
| Professional Fights*Weight[Light] | 0 (Ref.) |  |  |  |  | 0 (Ref.) |  |  |  |  |
| Professional Fights*Weight[Middle] | 0.01 | 0.08 | 41 | 0.07 | 0.943 | -0.05 | 0.20 | 90 | -0.27 | 0.787 |
| Professional Fights*Weight[Heavy] | 0.13 | 0.09 | 41 | 1.49 | 0.144 | -0.09 | 0.20 | 90 | -0.44 | 0.664 |
|  |  |  |  |  |  |  |  |  |  |  |
| **Reaction time (CNS Vital Signs)** | **Boxers** |  |  |  |  | **MMA Fighters** |  |  |  |  |
| **Predictors** | **Estimates** | **SE** | **df** | **t** | **p** | **Estimates** | **SE** | **df** | **t** | **P** |
| Weight[Light] | 0 (Ref.) |  |  |  |  | 0 (Ref.) |  |  |  |  |
| Weight[Middle] | 2.49 | 14.23 | 41 | 0.18 | 0.862 | 2.00 | 29.29 | 90 | 0.07 | 0.946 |
| Weight[Heavy] | 11.60 | 16.90 | 41 | 0.69 | 0.496 | 0.09 | 30.67 | 90 | 0.00 | 0.998 |
| Professional Fights | -0.21 | 0.48 | 41 | -0.45 | 0.656 | 1.40 | 2.39 | 90 | 0.59 | 0.560 |
| Professional Fights*Weight[Light] | 0 (Ref.) |  |  |  |  | 0 (Ref.) |  |  |  |  |
| Professional Fights*Weight[Middle] | 0.70 | 0.62 | 41 | 1.11 | 0.272 | -0.52 | 2.43 | 90 | -0.21 | 0.831 |
| Professional Fights*Weight[Heavy] | 0.51 | 0.70 | 41 | 0.73 | 0.468 | -0.51 | 2.45 | 90 | -0.21 | 0.836 |
|  |  |  |  |  |  |  |  |  |  |  |
| **Verbal memory (CNS Vital Signs)** | **Boxers** |  |  |  |  | **MMA Fighters** |  |  |  |  |
| **Predictors** | **Estimates** | **SE** | **df** | **t** | **p** | **Estimates** | **SE** | **df** | **t** | **P** |
| Weight[Light] | 0 (Ref.) |  |  |  |  | 0 (Ref.) |  |  |  |  |
| Weight[Middle] | 0.18 | 0.74 | 39 | 0.24 | 0.812 | -0.06 | 1.23 | 89 | -0.05 | 0.963 |
| Weight[Heavy] | -0.21 | 0.93 | 39 | -0.23 | 0.823 | 0.11 | 1.29 | 89 | 0.09 | 0.932 |
| Professional Fights | -0.04 | 0.03 | 39 | -1.75 | 0.088 | -0.02 | 0.10 | 89 | -0.23 | 0.822 |
| Professional Fights*Weight[Light] | 0 (Ref.) |  |  |  |  | 0 (Ref.) |  |  |  |  |
| Professional Fights*Weight[Middle] | 0.01 | 0.03 | 39 | 0.18 | 0.859 | 0.03 | 0.10 | 89 | 0.26 | 0.793 |
| Professional Fights*Weight[Heavy] | 0.05 | 0.04 | 39 | 1.39 | 0.172 | 0.00 | 0.10 | 89 | 0.00 | 0.999 |
